# A density-based approach for detecting complexes in weighted PPI networks by semantic similarity

**DOI:** 10.1371/journal.pone.0180570

**Published:** 2017-07-12

**Authors:** HongFang Zhou, Jie Liu, JunHuai Li, WenCong Duan

**Affiliations:** School of Computer Science and Engineering, Xi'an University of Technology, Xi’an, China; Texas A&M University College Station, UNITED STATES

## Abstract

Protein complex detection in PPI networks plays an important role in analyzing biological processes. A new algorithm-DBGPWN-is proposed for predicting complexes in PPI networks. Firstly, a method based on gene ontology is used to measure semantic similarities between interacted proteins, and the similarity values are used as their weights. Then, a density-based graph partitioning algorithm is developed to find clusters in the weighted PPI networks, and the identified ones are considered to be dense and similar. Experimental results demonstrate that our approach achieves good performance as compared with such algorithms as MCL, CMC, MCODE, RNSC, CORE, ClusterOne and FGN.

## Introduction

Empirical studies and theoretical modeling of networks have been studied for many years, and some relevant techniques have also been improved [1]. In addition to these, some of them have been applied to molecular biology successfully [2–4]. Proteins in biological system interact with each other by the PPI between them to implement various essential molecular processes. The complex biological system that is composed of proteins and Protein-Protein Interaction networks can be described formally as an undirected graph. In PPI networks, proteins are represented as nodes and the interactions are represented as edges [4]. By aid of PPI networks, we can obtain invaluable help in understanding the structures and features of molecular biosystems, such as protein complexes [2] and hub proteins in PPI networks.

The judgment of interactions between two proteins is generally based on the experimental methods. However, these methods are not always reliable [5], which means that the interaction networks may contain false positive edges. Due to the technical limitation, the experimental method cannot evaluate the strength of each interaction quantitatively and accurately [6]. Therefore, confidence weights of interactions should be taken into account by some certain computational approaches [7]. In fact, some relative computational approaches have been constantly applied to complement existing experimental approaches, such as gene neighborhood [8]. And most approaches are based on the similarities of protein attributes. To measure and visualize the functional similarities of gene products based on the existing annotation, several methods have been proposed and used to address the critical needs basically [9–10]. The similarity values can be used as weights of the edges and the PPI networks can be converted into a weighted graph. The related experimental results for extracting dense modules in weighted graph reveal that using biological information can improve the accuracy of protein complexes identification[11–13]. CMC [14] method assigns the reasonable weights to the corresponding interacting protein pairs, and the weight is the therein interaction dependability. This algorithm can form as many as possible large clusters in the protein networks, and then delete highly overlapping ones. CFinder [15] is a popular published overlapping clustering method. And this method can determine functional modules in PPI networks. ClusterOne [16] is mainly used to find overlapping proteins in PPI networks. It has a good performance in the yeast data. PEWCC [17] is a graph-based clustering algorithm for protein complex identification. It can be divided into two steps, the first step is calculating the therein reliabilities, and the second is predicting protein complexes by weighted clustering coefficients. FGN [18] combines GO annotations and GO semantic structures to decide the corresponding protein semantic similarity. First, the protein semantic similarity is calculated according to their predetermined GO annotations. Second, the expending RRW algorithm is used to extend attachment proteins to the cores. The graph-based approaches can remove doubtable interactions before clustering, so that FGN can identify protein complexes more successful. GMFTP [19] is an algorithm that can identify overlapping and individual proteins. A model is created by the function of the protein nodes and its topological properties in the networks, which describe the generation and functional characteristics of the protein interaction networks. Experimental results indicate that GMFTP can effectively identify overlapping protein complexes in PPI networks. WPNCA [20] is a novel algorithm based on the core attachment structure of protein complexes with its neighboring nodes. Firstly, they proposed a weighting algorithm based on the probability of adjacent nodes, and then divided the protein networks into several dense clusters. Experiments were performed on the four datasets. From the relative experimental results, it can be found that WPNCA is a successful one in detecting complexes. DCAFP [21] presents a new way to identify complexes. It first defines the concept of each protein preference vector because preference vector can represent the functional category of the protein complex. DCAFP combines preference vector with network topology to improve the accuracy of protein complex recognition. DUC [22] algorithm builds a protein interaction network as a model. Considering such traditional algorithms ignore the adjacent information in the networks. DUC integrated the expected densities and degrees. The experimental results show that such model provides a new insight for the identification of protein complexes. EGCPI [23] algorithm is a traditional graph clustering one, in which the similarity between proteins are referred by gene ontology database. And the complexes are found by the homogeneity of the properties. RFC [24] is a fuzzy clustering algorithm, in which it establishes the fuzzy relationship between proteins and transforms it into some certain equivalence relation. This method can identify overlapping proteins. DyCluster[25] proposed a framework to model dynamic protein networks, it first construct a framework to identify protein complexes, and then detects complexes by clustering in a dynamic networks.

The existing methods based on the topology of protein-protein interaction network and biological information have inspired us to improve the accuracy of protein complex recognition. In order to solve the problem caused by false positive and false negative data effectively, we proposed a new method-DBGPWN, which combined the density and semantic similarity in PPI networks. In this paper, we first introduce the semantic similarity and construct a weighted PPI networks. Then, a new concept of semantic clustering coefficient is proposed, which is used for detecting protein complexes in the PPI networks.

We performed experiments on four different protein-protein interaction networks, which are widely used in biological experiments. Experimental results demonstrate that DBGPWN can identify more functional protein complexes and improve the accuracy of protein complexes prediction.

The remaining part of the paper is organized as follows. Section 2 (Material and Method) introduces the new algorithm-DBGPWN. Section 3 (Result and Discussion) is the detailed descriptions and analysis of the experiments. Finally, the fourth part (Summary) is the conclusion.

## Material and method

### Semantic similarity

**Fig 1:**
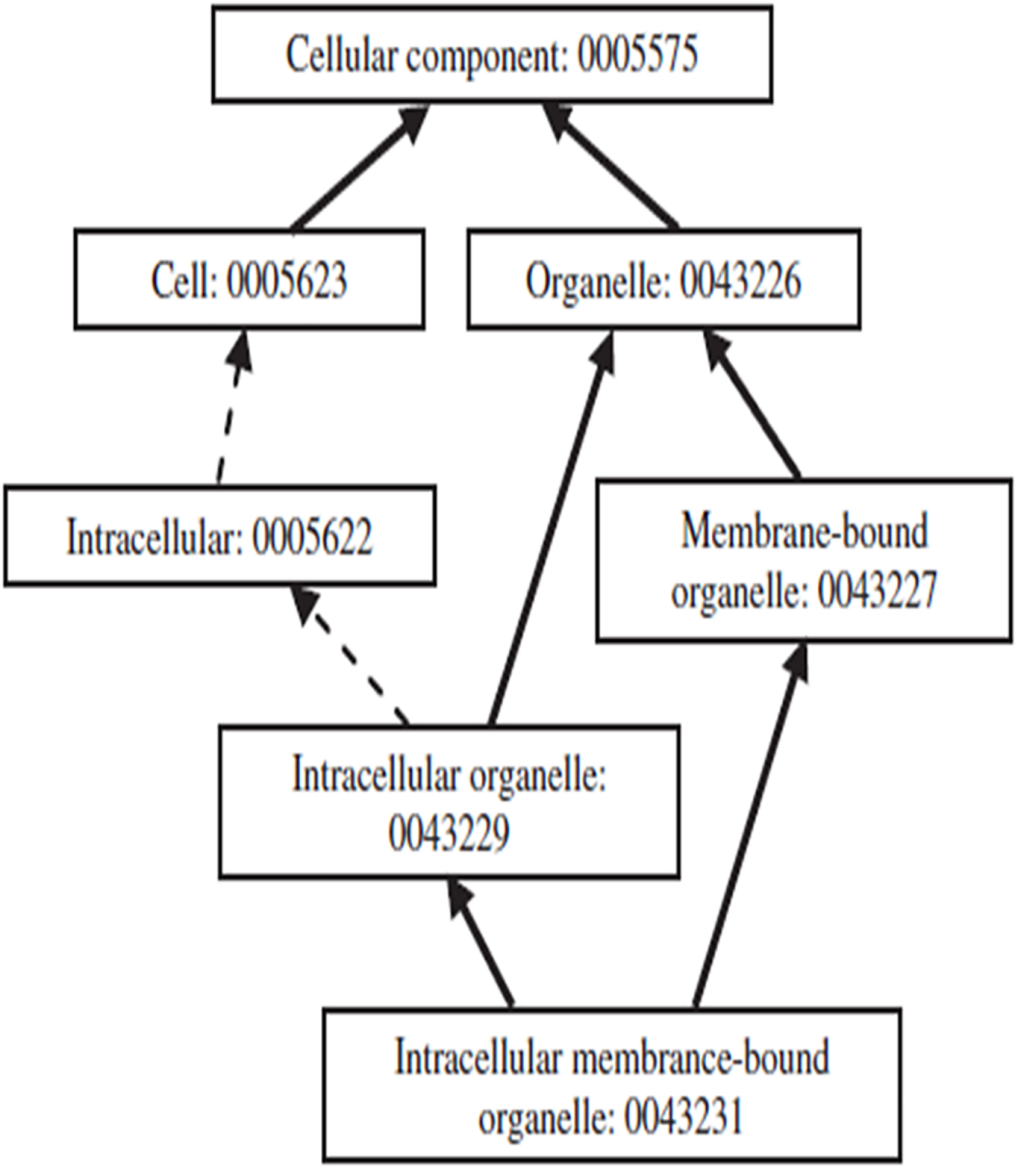
A united DAG.

We put forward a new concept called as Unit Similarity Measure, in which GO terms annotating proteins are regarded as a semantic collection, and their corresponding DAGs are merged into one united DAG (Directed Acyclic Graph). DAG is a method to represent the structure of gene ontology database. In a DAG, attributes are represented by nodes, and the semantic relations are expressed by edges. Gene Ontology is a large collaborative public bioinformatics database, whose founders’ aim is to unify the representation of gene and gene product attributes across all species [9]. GO includes two kinds of semantic relations, which are represented by ‘is-a’ and ‘part-of’. The marks ‘is-a’ and ‘part-of’ represent a class-subclass relation and a partial ownership relation respectively. GO contains amounts of biological or biochemical terms for describing gene products based on their functions or locations in the cell. All the terms can be classified into three kinds, which are biological process, cellular component and molecular function respectively. For example, P56524 is annotated by several GO terms (GO: 0008134, GO: 0005515, GO: 0019901, GO: 0030955 and GO: 0033613), and their relations can be modeled as a united DAG, as shown in Fig 1.

The black arrows point to five GO terms annotating protein P56524 and the main function they represent is binding (Fig 1). So the functional similarity between two proteins can be measured by comparing the united DAGs of their annotations. If a protein has several annotations about binding, it can be considered to be functional similar to the protein P56524.

**Definition 1** (*S-*value) Given a set of terms united DAG_*A*_ and a set of GO terms, its *S-value* related to term *A* for any term *t* in *T*_*A*_ is shown in Eq 1 if *A* ⊂ *T*_*A*_.

$$S_{A}(t)=\{\begin{array}{cc} 1 & t=A\\ \max\{w_{e}\cdot S_{A}(t^{\prime })|t^{\prime }\in childrenof(t)\} & t\neq A \end{array}$$ (1)

Where *w*_*e*_ is the semantic contribution weight of the edge e (*e* ∈ *E*_*A*_) which links term *t* with its child term *t*', the optimal value of *w*_*e*_ for ‘is-a’ and ‘part-of’ relations are 0.8 and 0.6 respectively[10]. The semantic similarity between two GO terms can be formally calculated based on the S-values of their ancestor terms.

**Definition 2** (Semantic Similarity) Given two proteins *a* and *b*, *A* is a set of GO terms annotating *a*, *B* is a set of GO terms annotating *b*, their DAGs can be represented as DAG_*A*_ = (*A*,*T*_*A*_,*E*_*A*_) and DAG_*B*_ = (*B*,*T*_*B*_,*E*_*B*_). *T*_*A*_ and *T*_*B*_ are the sets of GO terms including their ancestor ones. The semantic similarity between these two proteins can be calculated according to Eq 2.

$$Sim(a,b)=\frac{\sum_{t\in T_{A}\cap T_{B}}\left(S_{A}(t)+S_{B}(t)\right)}{\sum_{t\in T_{A}}S_{A}(t)+\sum_{t\in T_{B}}S_{B}(t)}$$ (2)

In Eq 2, *S*_*A*_(*t*) is the S-value of the term *t* according to DAG_*A*_, and the *S*_*B*_(*t*) is the S-value of the terms t according to DAG_*B*_.

**Definition 3** (Harmonic Semantic Similarity) The quadratic mean of three semantic similarities is regarded as the measure of judging whether two proteins are semantic similar. The calculation formula can be expressed as Eq 3.

$$HSim(a,b)=\sqrt{\frac{Sim_{p}{\left(a,b\right)}^{2}+Sim_{f}{\left(a,b\right)}^{2}+Sim_{c}{\left(a,b\right)}^{2}}{3}}$$ (3)

where *Sim*_*p*_(*a*,*b*), *Sim*_*f*_(*a*,*b*) and *Sim*_*c*_(*a*,*b*) represent three kinds of semantic similarities-*biological process*, *molecular function* and *cellular component*.

### Density description

The basic idea of DBGPWN is analogous to the classical clustering algorithm—DBSCAN [22]. DBSCAN expands regions with significantly high density into cluster and discovers clusters with arbitrary shapes in spatial database with noises. And it can find the arbitrary shape clusters. A cluster is defined as a maximal set of density-connected points. Basically, a point *q* is directly density-reachable from a point *p* if the distance between them is smaller than a given distance *ε*, and the point *q* has enough neighboring points around it. The point *q* is considered to be density-reachable from the point *p* provided that there is a sequence of points *p*_1_,…,*p*_*n*_ (*p*_1_ = *p* and *p*_*n*_ = *q*) where each *p*_*i*+1_ is directly density-reachable from *p*_*i*_. *q* and *p* are density-connected to each other if they are both density-reachable from another one. It is noticed that density-reachability is an asymmetric relation, but density-connectivity is a symmetric one.

Generally, two objects in the same cluster may have several common neighbors. For instance, more common friends two persons have, more likely they belong to the same community in social networks. If two data points are density-reachable and have several common neighbors, they are more likely to be a cluster. In addition, it is required to adjust two parameters (*MinPts* and *ε*) in DBSCAN, but it is always hard to predetermine their values. Therefore, DBGPWN is proved to be more suitable in weighted networks although it is proposed based on the basic idea of DBSCAN. The new algorithm determines dense subgraphs in weighted PPI networks. The basic idea of DBGPWN is to expand regions with significantly high density into cluster and discover clusters with arbitrary shapes in spatial database.

**Definition 4** (Directly Density-reachable) Given a PPI network weighted by semantic similarity *G*(*E*,*V*,*W*), two proteins *i* ∈ *V* and *j* ∈ *V*, a parameter *θ* ⊂ [0,1], the proteins *i* and *j* are defined to be density-reachable directly if *SCC*(*i*,*j*) ≥ *θ*.

**Definition 5** (Density-reachable) Given a PPI network weighted by semantic similarity *G*(*E*,*V*,*W*), two proteins *i* ∈ *V* and *j* ∈ *V*, the proteins *i* and *j* are defined to be density-reachable provided that there is a sequence *p*_1_,…,*p*_*n*_ (*p*_1_ = *i* and *p*_*n*_ = *j*) of proteins in *V* where *p*_*i*+1_ and *p*_*i*_ are directly density-reachable.

**Definition 6** (Density-connected) Given a PPI network weighted by semantic similarity *G*(*E*,*V*,*W*), two proteins *i* ∈ *V* and *j* ∈ *V*, the proteins *i* and *j* are defined to be density-connected provided that there is a protein *k* which is both density-reachable to *i* and *j*. In addition, if *i* and *j* are directly density-reachable without a third protein being directly density-reachable to them, *i* and *j* are still defined to be density-connected.

**Fig 2:**
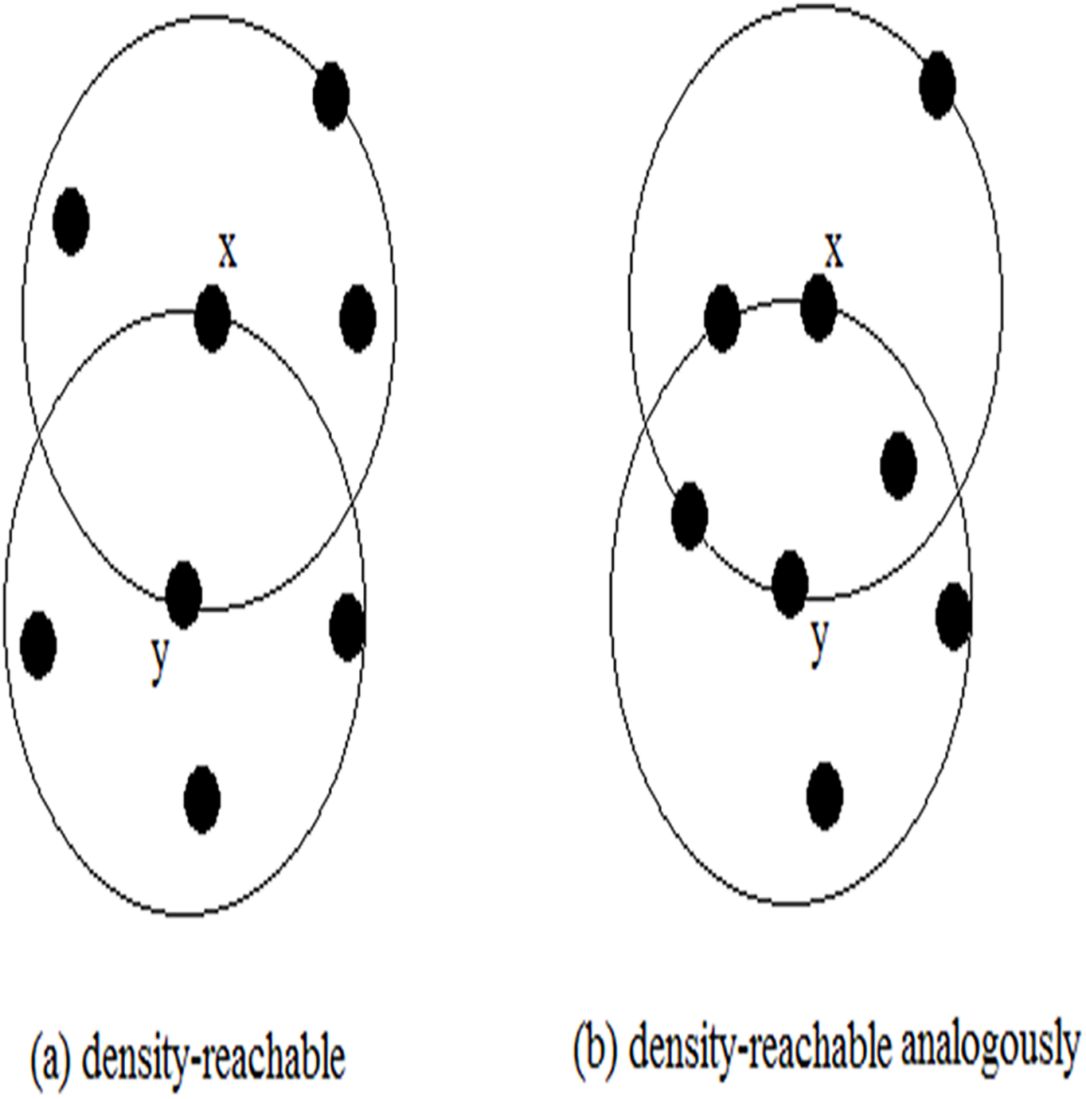
Two cases of directly density-reachable.

These definitions can be modified to make clustering more accurately. If *MinPts* = 3, the points *x* and *y* are both directly density-reachable to each other on two cases respectively, as shown in Fig 2. However, *x* and *y* should be identified to be more similar on the condition (b) in Fig 2, as they have more common density-reachable points than that on the condition (a) in Fig 2.

Two points *x* and *y* both have four *ε*-*neighbours*, the difference is the number of common neighbors(Fig 2). Generally, two objects in a same cluster may have several common neighbors. If two data points are density-reachable and have several common neighbors, they are more likely to be the part of a cluster. In addition, it is required to adjust two parameters (*MinPts* and *ε*) and parameters setting is usually hard to be determine in advance. Therefore, we propose a new measure of directly density-reachable. Its fundamental principles are analogous to edge-clustering coefficient.

For a PPI network weighted by semantic similarity, this measure is essentially a calculation of biological similarity based on the network topology, so it can be called as semantic clustering coefficient defined as following.

**Definition 7** (Semantic Clustering Coefficient) Given a PPI network weighted by semantic similarity *G*(*E*,*V*,*W*), and its adjacency matrix *A*_*i*,*j*_(, *A*_*i*,*j*_ equals to the weight *W*_*i*,*j*_ of this edge if there is an edge between nodes *i* and *j* in G, else it equals to 0; *A*_*i*,*j*_ = 1 if *i* = *j*), the semantic clustering coefficient between two proteins *i* and *j* is represented as Eq 4.

$$SCC(i,j)=\frac{\left(\sum_{k\in V}\left\{A_{i,k}+A_{k,j}|A_{i,k}>0\&A_{k,j}>0\right\}\right)-2}{\sum_{k\in V}A_{i,k}+\sum_{k\in V}A_{k,j}}$$ (4)

If there are several proteins being semantic similar to both proteins *i* and *j*, and the protein *i* is semantic similar to the protein *j* as well, *SCC*(*i*,*j*) may be high. Essentially *SCC*(*i*,*j*) depends on the number of the high weighted triangles containing *i* and *j*. In a PPI network, their intra-interactions may be high if a protein in a complex has more interactions with others. Therefore, a complex may contain many triangles with high weighted interactions in a weighted PPI network.

**Fig 3:**
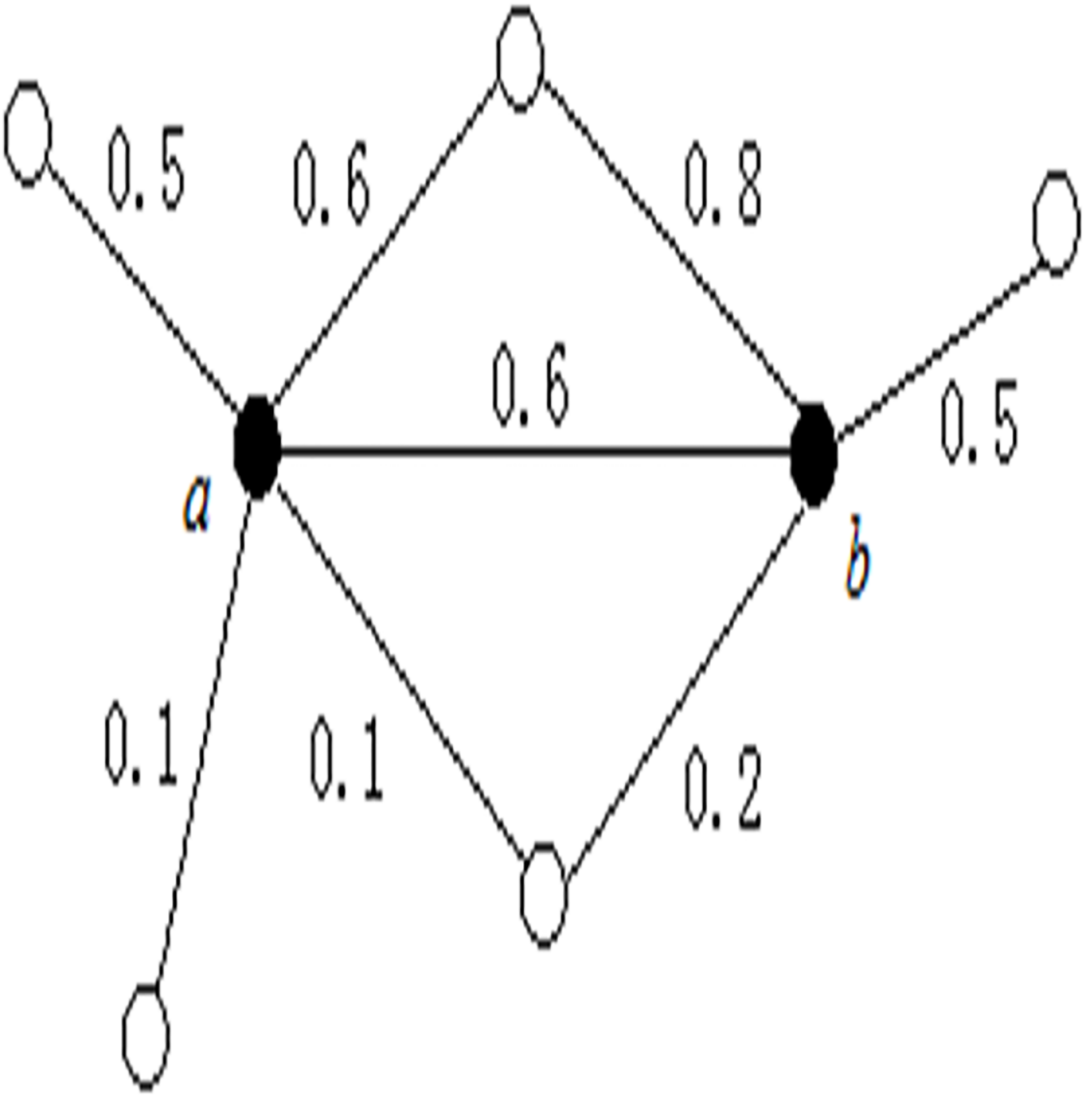
Clustering property in weighted networks.

As shown in Fig 3, it shows several proteins and their interactions with supposed weights.

The points *a* and *b* show an obvious clustering feature because their three high weighted interactions form a triangle. Based on Eq 4, *SCC*(*a*,*b*) = 0.725. The semantic clustering coefficient of two proteins is applied to determine whether they are directly density-reachable in DBGPWN, and it can be regarded as the probability value if they belong to the same complex.

In the DBSCAN algorithm, the parameters *ε* and MinPts represent the given minimum distance and the number of nodes within a given neighborhood respectively. Our proposed DBGPWN algorithm contains only one parameter, and in DBGPWN algorithm, two nodes are defined to be directly reachable by density if their semantic coefficient *SCC* is equal to or greater than the parameter *θ*. That is to say, these two protein nodes are regarded to be connectable. To explain the DBGPWN algorithm more explicitly, the pseudo-code is shown in Table 1.

**Table 1:** The Pseudo-code of DBGPWN algorithm.

**Algorithm** DBGPWN
**Input:** A weighted PPI network *G*(*E*,*V*,*W*), a parameter *θ*; **Output:** Clusterings *C*_1_,…,*C*_*n*_;
**Begin:** 1. calculate semantic clustering coefficient between the connected proteins; 2. Let *i*, *j* is two proteins; 3. **For** (*i* = 0; *i*<*V*; *i*++) 4. Calculate the *SCC* of each proteins 5. **if** (protein *i* do not belong to any cluster) **then** 6. create a new cluster *C*; 7. **For** (*j* = 0; *j*<*V*; *j*++) 8. Compare the *SCC* of proteins 9. **if** (protein *j* do not belong to any cluster) **then** 10. **if** (*i* is density-connected to *j*) **then**
11. insert protein *j* into *C*; 12. **End For** 13. **End for** **End.**

## Results and discussion

### Experimental data

In the experiments, four popular datasets-Gavin, DIP, Krogan and MIPS are used to verify our proposed DBGPWN algorithm.

Gavin and DIP were used to construct the network. The Gavin dataset consists of 1430 proteins and 6531 interactions, which is a relatively dense and small-scale protein network. The DIP dataset (20091230 version) consists protein information, interaction confidence and experimental techniques for detecting interactions, which constitute a network of relatively sparse large-scale protein networks. And it contains 4930 proteins and 18693 interactions. Krogan and MIPS consist of 3581 proteins, 14077 interactions and 4546 proteins, 12317 interactions respectively.

### Evaluation metrics

To measure the comparability between predicted clusters and known complexes, we employ the most widely evaluation metrics used in experiments. Their related definitions are described below.

**Definition 8** (Overlapping Score) Given a predicted cluster *P* and a known complex *K*, the Overlapping Score between *P* and *K* is defined as follows.

$$OS(P,K)=\{\begin{array}{cc} \frac{\left|V_{P}\cap V_{K}\right|}{\left|V_{P}|\cdot |V_{K}\right|} & |V_{P}\cap V_{K}|\neq 1\\ 0 & |V_{P}\cap V_{K}|=1 \end{array}$$ (5)

where |*V*_*P*_ ∩ *V*_*K*_| is the sum of the common proteins in the predicted cluster *P* and the known complex *K*, |*V*_*P*_| is the size of the predicted cluster and |*V*_*K*_| is the size of the known complex.

Sensitivity and specificity are two widely used measures for evaluation algorithm performance.

**Definition 9** (Sensitivity and Specificity) Let *TP* (True Positive) represents the number of the predicted clusters matched with the known complexes when *OS*(*P*,*K*) ≥ *σ*, *FP* (False Positive) equals the total number of the predicted clusters minus *TP*, and *FN* (False Negative) represents the number of the known complexes which are not matched with the predicted clusters. Then, sensitivity (*S*_*n*_) and specificity (*S*_*p*_) can be respectively expressed as follows.

$$S_{n}=\frac{TP}{TP+FN}$$ (6)

$$S_{p}=\frac{TP}{TP+FP}$$ (7)

Sensitivity is the fraction of the true-positive predictions out of all the true ones, and specificity is the fraction of the true-positive predictions out of all the positive ones[13]. To make a comprehensive comparison, F-measure is used as an evaluation metric which is a comprehensive metric combined sensitivity and specificity. It can be formally represented as Eq 8.

$$F-measure=\frac{2\cdot S_{n}\cdot S_{p}}{S_{n}+S_{p}}$$ (8)

Moreover, we employ the *p-vlaue* to measure the biological relevance of the returned clusters and the ability of a method in term of clustering proteins.

**Fig 4:**
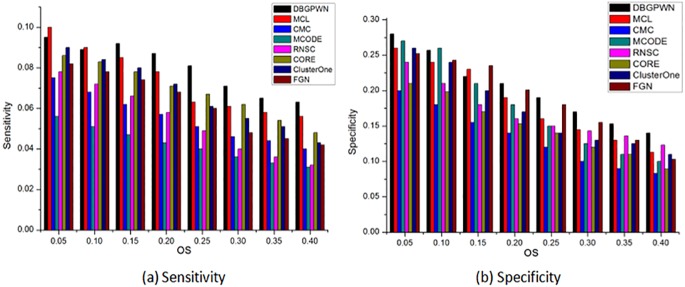
Performance comparisons on the Gavin.

**Definition 10** (*p-vlaue*) Given a cluster of size *n* with *m* proteins sharing a common annotation *x*, then the probability of observing *m* or more proteins annotated with *x* out of those *n* proteins is defined as p-value.

$$p\text{-}value=\left\{\sum_{i=m}^{n}\frac{C_{M}^{i}\cdot C_{N-M}^{n-i}}{C_{N}^{n}}|i<M\wedge m>1\right\}$$ (9)

where *N* is the number of proteins in the database with *M* of them sharing annotation *x*. Thus, the lower the *p-value* is, the more significant for representation the associated GO term *x* is. Generally, the recommended cutoff value of *p-value* to distinguish significant from insignificant groups is 0.05. **Definition 11** (MMR) MMR (Maximum Matching Ratio) is a maximal matching measure in a bipartite graph. The two sets of nodes in the graph represent the references and predicted complexes. An edge which connects the reference complex and the predicted one is weighted by the corresponding overlap score. We compare DBGPWN with MCL[26], CMC[13] MCODE[27], RNSC[28] CORE[29], ClusterOne[16] and FGN in our paper. To make a reasonable comparison, we run DBGPWN on the networks of proteins dataset in which the interactions have been weighted by Unit Similarity Measure. All the protein complexes identified are compared with standard known complexes. The performance of each method is evaluated in terms of sensitivity, specificity and F-measure. DBGPWN achieves good performance on the Gavin data set (Fig 4). From Fig 4(A), we can see that the DBGPWN has obvious advantage when *OS* ≥ 0.2. The value of sensitivity in DBGPWN is twice as high as that in MCODE and RNSC algorithms. Therefore, DBGPWN has a good experimental effect on the Gavin dataset compared with other seven algorithms.

**Fig 5:**
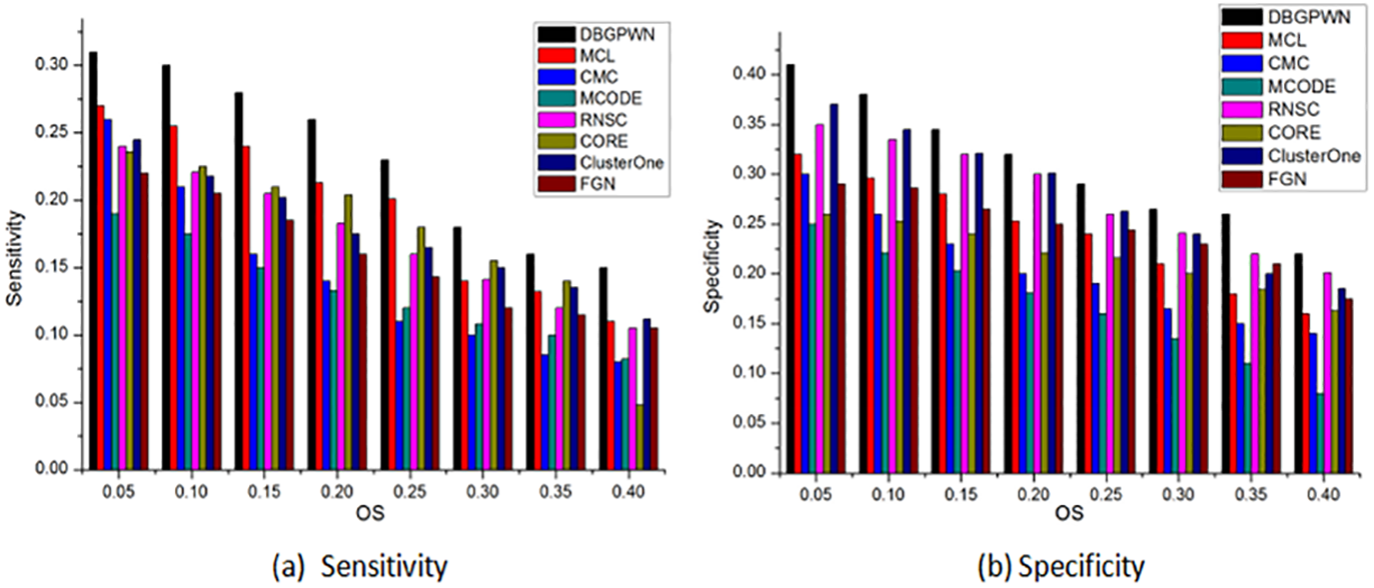
Performance comparisons on the DIP.

The experimental result shows that DBGPWN is more effecitve than other algorithms on the DIP data set (Fig 5). DBGPWN has achieved the best results compared to other algorithms in the aspects of sensitivity or specificity. The protein complexes identified by DBGPWN on this dataset is more accurate as shown in above.

**Fig 6:**
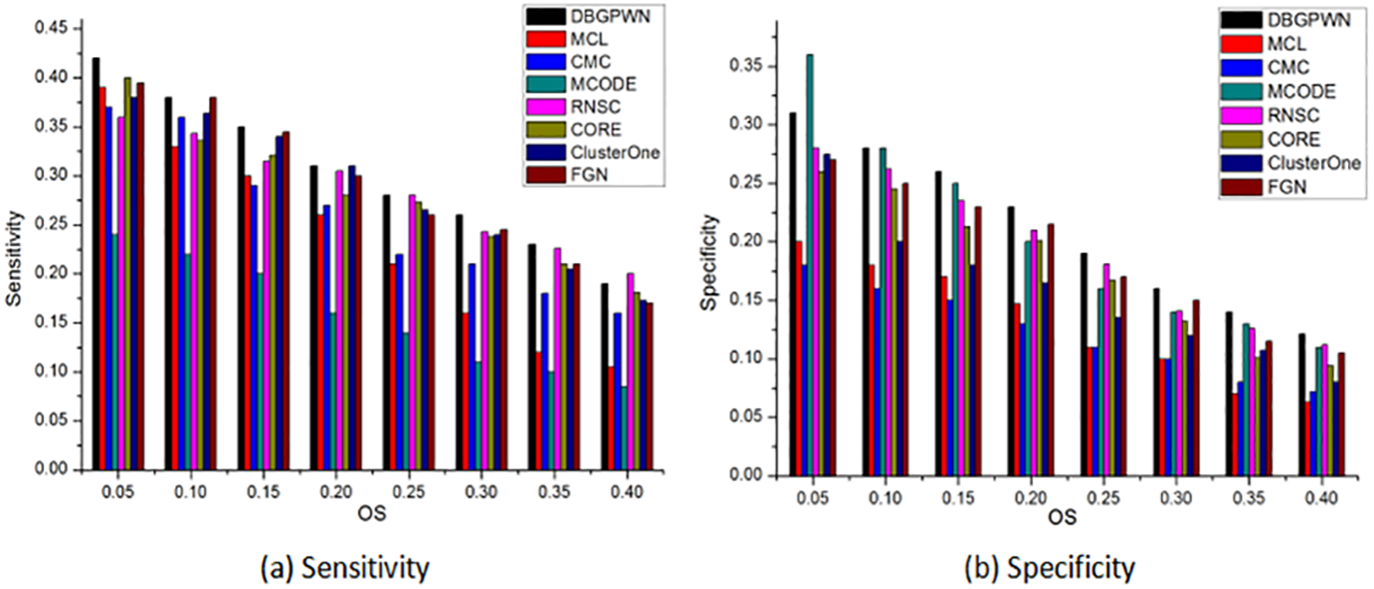
Performance comparisons on the Krogan.

We can see that the DBGPWN algorithm has not achieved good performance on the Krogan data set (Fig 6). The sensitivity values of RNSC and CORE algorithms are higher than that of DBGPWN when *OS* = 0.25. MCODE is the most effective method when *OS* = 0.5. But DBGPWN shows good performance in other *OS* value. So it is still advantage good one as compared with than other contrast methods.

**Fig 7:**
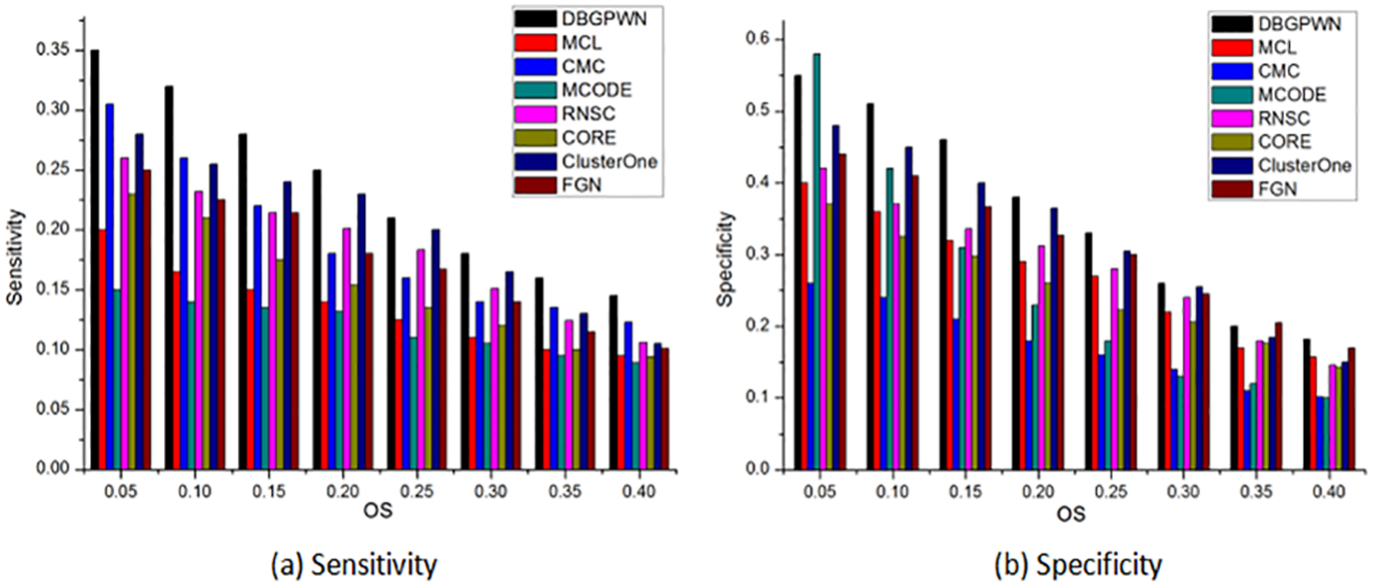
Performance comparisons on the MIPS.

DBGPWN has achieved good experiment result in terms of sensitivity (Fig 7). Its value is higher than other contrast methods. The specificity of DBGPWN is slightly lower than that of MCODE when *OS* = 0.05. But the former still is the best effective algorithm in sensitivity and specificity metrics. Its specificity is twice times higher than that of CMC and MCODE.

**Fig 8:**
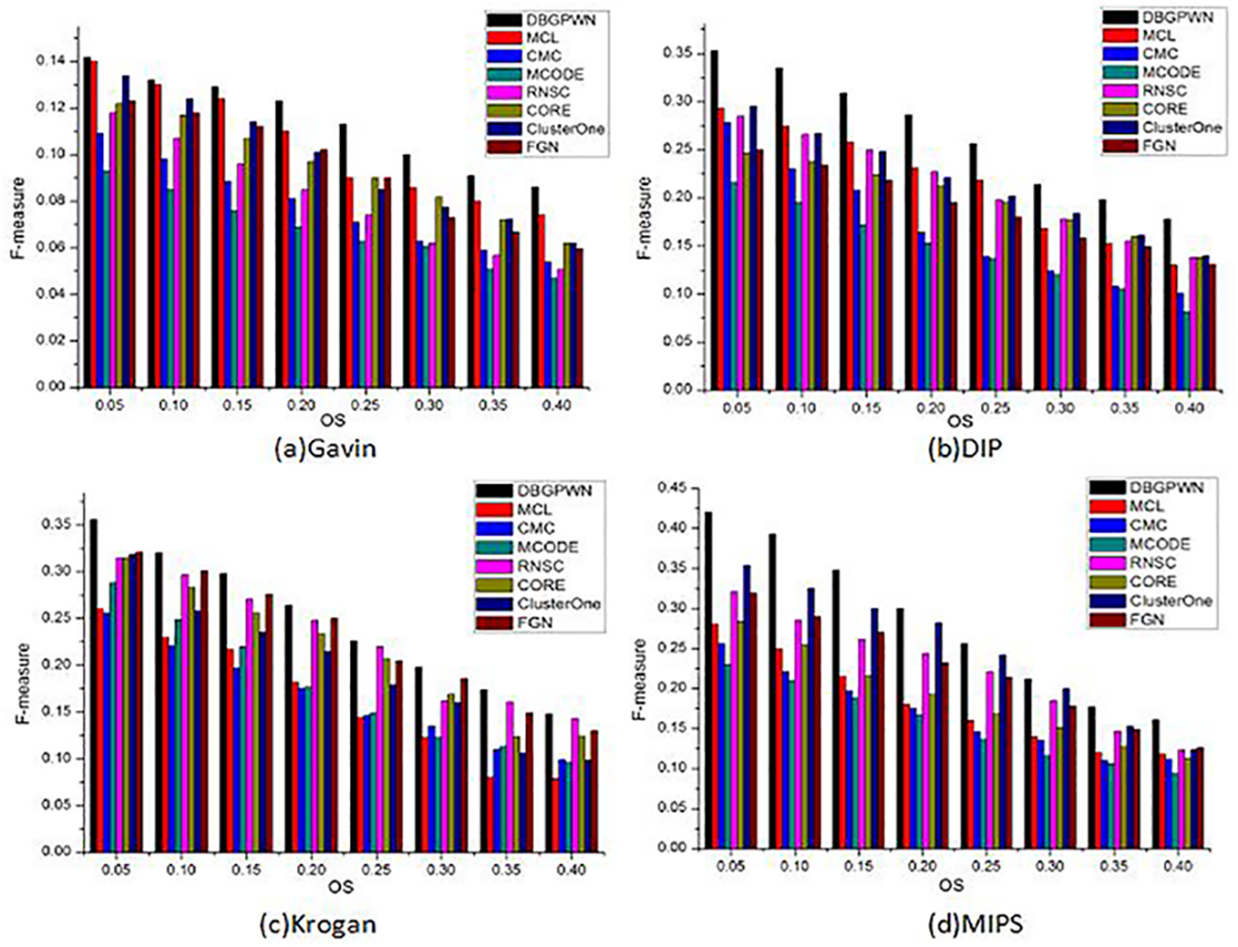
F-measure performance comparisons on the four datasets.

F-measure is a comprehensive metric. It can describe the merit of the experiment results in details (Fig 8). In Fig 8(A), we can get the following inclusions. When *OS* < 0.15, DBGPWN algorithm has not gotten an effective result. But with the OS value increases, DBGPWN shows obvious advantages. Meanwhile, as shown in Fig 8(C), the F-measure of DBGPWN and RNSC algorithms are not perfect, but the experimental results of those two methods are better than other ones. In Fig 8(B) and 8(D), it can be seen that DBGPWN is an effective method.

We present two clusters returned by our method with low p-values, and they are well matched with known complexes. DBGPWN groups a cluster which contains 11 proteins; this cluster is matched with a known protein complex in the Gavin. The 6 proteins in the cluster are annotated with a common GO term DNA repair (GO: 0006281) of biological process, and the corresponding p-value is 6.68e-15. In addition, there are 6 proteins sharing another biological process cellular response to DNA damage stimulus (GO: 0006974), and the corresponding p-value is 2.89e-14.

DBGPWN also finds a high quality cluster with 6 proteins (YDL225W, YDR168W, YCR002C, YJR076C, YLR314C, and YHR107C), and 5 out of them can be discovered in a known complex *Cytoskeleto septin filaments* (YDL225W, YDR218C, YGR059W, YCR002C, YJR076C, YLR314C, and YHR107C). For the molecular *function nucleotide binding* (GO: 0000166) annotating 5 proteins in the cluster, the *p-value* is 0.00027. For the *molecular function guanosine triphosphate binding* (GO: 0005525) shared with 5 proteins, the *p-value* is 9.1e^-9^. Moreover, 4 proteins perform the *molecular function structural molecule activity* (GO: 0005198), and the *p-value* of the cluster is 6.04e^-9^.

**Fig 9:**
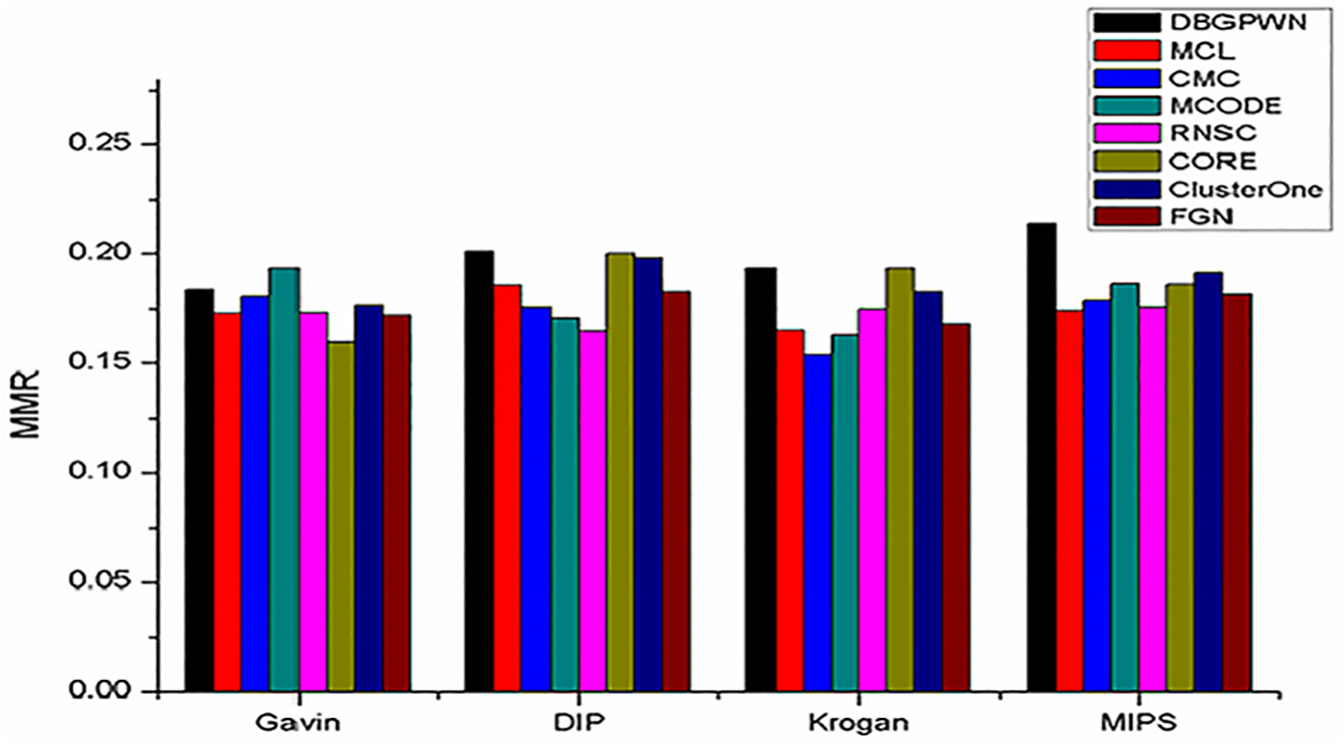
MMR performance comparisons on the four datasets.

MMR shows the matching rate of protein complexes on the different datasets (Fig 9). We can see that the DBGPWN performs slightly worse than the MCODE method on the Gavin dataset, but it is much better than other ones on the MIPS dataset. Although the experimental results are not same on different datasets, we can judge that DBGPWN is more effective in identifying protein complexes than other four contrast algorithms.

## Conclusions

In the paper, we made the following two contributions. Firstly, a simplified semantic similarity measurement is used to measure the strength of each interaction in PPI networks. Secondly, a new density-based algorithm is proposed to search for the dense regions in the weighted PPI networks. And the proteins coupled tightly are classified into the same cluster. The experimental results demonstrate that DBGPWN has a good clustering performance.(i) DBGPWN does not require any auxiliary information, and it is not sensitive to the input parameter.(ii) Compared with MCL, CMC, MCODE, RNSC, CORE, ClusterOne and FGN, DBGPWN can get more accurate protein complexes.

## Supporting information

S1 Table Protein datasets used in experiment. (DOCX)
