# Supplementary material for: A density-based approach for detecting complexes in weighted PPI networks by semantic similarity
Source: PLoS One. 2017 Jul 12;12(7):e0180570. doi: 10.1371/journal.pone.0180570 (PMC5507511; doi:10.1371/journal.pone.0180570)
Supplement: S1 Table — (DOCX) [file pone.0180570.s001.docx]

**S1 Table. Protein datasets used in experiment**

| Dataset | Proteins | Interactions |
| --- | --- | --- |
| Gavin | 1430 | 6531 |
| DIP | 4930 | 18693 |
| Krogan | 3581 | 14077 |
| MIPS | 4546 | 12317 |
